# On frogs, toxins and true friendship: an atypical case report

**DOI:** 10.1186/s40409-016-0057-8

**Published:** 2016-01-25

**Authors:** Cláudio Tadeu Daniel-Ribeiro, Christian Roussilhon

**Affiliations:** Laboratório de Pesquisa em Malária, Instituto Oswaldo Cruz, Fundação Oswaldo Cruz, Pavilhão Leônidas Deane, 5º andar, Av. Brasil, 4365, CEP 21045-900 Rio de Janeiro, RJ Brasil; Unité de Génétique Fonctionnelle des Maladies Infectieuses, Institut Pasteur and Centre National de la Recherche Scientifique, Unité de Recherche Associée 3012, Paris, France

**Keywords:** Dart frogs, Dendrobatidae, Toxins, French Guiana

## Abstract

The authors report a series of events including the scientific interest for poisonous dendrobates of French Guiana, the human confrontation with the immensity of the evergreen rainforest, the fragility of the best-prepared individuals to a rough life, and the unique and very special manifestation of a solid friendship between two experts and enthusiasts of outdoor life. In the evergreen forest of South America, as in many other scientific field expeditions, everything may suddenly go wrong, and nothing can prepare researchers to accidents that may occur in a succession of uncontrollable errors once the first mistake is done. This is what happened during an expedition in search for dendrobates by an experienced forest guide and naturalist. The authors decided to report the story, considering that it deserved to be brought to the attention of those interested in venomous animals and toxins, in order to illustrate the potential danger of dealing with these organisms.

*"If you want to go fast, go alone...if you want to go far, go together" African proverb*

## Background

We take the opportunity of using a scientific journal such as the prestigious *Journal of Venomous Animals and Toxins including Tropical Diseases (JVATiTD)* to tell the readers an amazing and true story that we have heard a few years ago. This narrative involves poisonous frogs, animal toxins and friendship, topics of potential interest to zoologists, and we wish to share it with other scientists. As far as we can trust our own memories, we managed to recount here the facts as precisely as we knew they happened (according to our records, between 1983 and 1984) and we tried, sometimes, to imagine and describe to the best the protagonist’s emotions and thoughts^1^. The authors would like to dedicate this text to Andrew and Rodolpho^2^.

A way to begin this story would be to describe a remarkable episode in Andrew’s life when he did his military service in the most remote areas of the Amazonian forest in French Guiana, South America. For the French man, young at that time, this was a unique and fascinating opportunity to discover how the indigenous people managed to survive in the inhospitable evergreen forest, where he had learnt to respect their elaborate survival knowledge as well as their strength, their courage and their endurance in a harsh environment. Once accepted into the indigenous community, he gradually learned to identify potentially poisonous mushrooms and fruits of the forest, and to use his knife in a way he never thought to be able before, to hunt and survive alone, avoiding dangerous animals and unnecessary risky situations. He gradually began to marvel, depending on the daily lives of the rainforest dwellers, at their unbeatable survival strategies and their respectful and intimate relation to the deep forest.

After several months sharing his everyday life with indigenous people, he developed strong relationships and became intimately connected with real friends as if he were a member of their families. He also understood – in a peaceful and serene way – that he was living an extraordinarily crucial and privileged experience that would permanently affect the rest of his life.

For most of the twenty-five years following his military service he became a regular visitor to the remote village in which he was virtually adopted. At the same time, in France, he was leading a classic professional life as an entrepreneur, but most of his colleagues and employees were completely unaware of this “double” life when he used to spend almost every single year a full month with his adoptive indigenous family.

During one of his stays in the forest village, Andrew met Rodolpho and he discovered in this man a strong and friendly character committed, as he was himself, to a simple and rustic lifestyle. Thereafter, they shared several opportunities to travel together in various remote forest areas of French Guiana, when living and hunting with local inhabitants. After several years of interaction, a mutual respect and a strong and authentic friendship developed between them.

Rodolpho is the man who was, years after their first meeting, involved in the almost fatal accident that we wish to report here and share with the readers.

## Case presentation

**Fig. 1:**
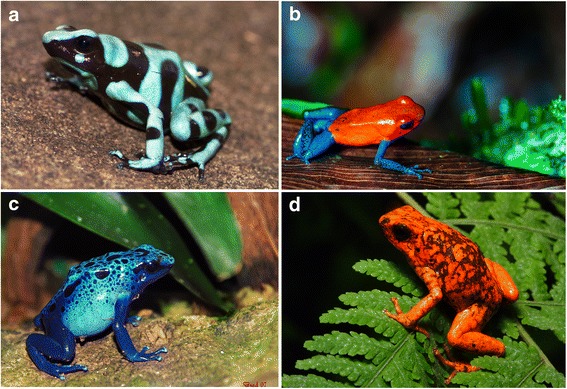
Examples of Dendrobatidae frogs. **a** *Dendrobates auratus*, also known as the green and black poison dart frog or the green and black poison arrow frog (“*Dendrobates auratus*, Bocas del Toro” by Brian Gratwicke, CC BY 4.0, https://flic.kr/p/ajE9io). **b** *Oophaga pumilio*, strawberry poison frog or strawberry poison dart frog (“Blue jeans frog” by Danel Solabarrieta, CC BY-SA 2.0, https://flic.kr/p/gRYCXh). **c** *Dendrobates azureus*, the blue poison dart frog or blue poison arrow frog (“Frog, *Dendrobates azureus*” by Brad Francis, CC BY-ND 2.0, https://flic.kr/p/4fx2Zx). **d** *Oophaga sylvatica* (“Little-devil poison frog, *Oophaga sylvatica* from Provincia Esmeraldas, Ecuador” by Santiago R. Ron-FaunaWebEcuador, CC BY-ND 2.0, http://zoologia.puce.edu.ec/vertebrados/anfibios/anfibiosecuador/default.aspx)

Rodolpho became a qualified and experienced professional naturalist guide in French Guiana, and he became involved in this activity with such enthusiasm and competence that his reputation crossed the Atlantic to reach ecotourists, environmentalists and scientists of Western Europe. One day, a group of Swiss zoologists hired him for a deep forest mission aimed at capturing the beautiful and elusive dart-poison frogs that present exuberant colors and produce a sticky secretion with very potent neurotoxins. The lethality of the different toxins of frogs in the family Dendrobatidae are not yet completely known, but a set of strong evidences point to batrachotoxins as the most toxic lipophilic alkaloid toxin secreted through the skin of these frogs, and one of the most toxic alkaloids on earth [1, 2]. Toxicity seems to be related to the color contrast [3] (Fig. 1), which operates as a strong warning, a kind of “do not even touch me” signal. The expression “dart frog” comes from the Native Americans’ use of the frog toxic secretions to poison the tips of blow darts. Rodolpho was asked to find some of these colorful frogs prevalent in Guiana for the zoologists.

He received the scientists with whom he went up the river for several hours, choosing on purpose a remote and uninhabited area, away from busy places. At short distance from the river, a base camp was set up, from where it was decided to go in search of the frogs that were to be captured alive for scientific purposes.

Unused to the walk in humid and hot forest conditions, the scientists were moving slowly and they soon started to show some difficulty in keeping up with Rodolpho’s agility. Somewhat annoyed by the slowness of the group of scientists who delayed his progress, and by their ignorance of the preferred habitats of the colorful frogs, Rodolpho strongly suggested that they return to the camp and wait for him while he would quickly look deeper in the locally dense forest. Without further explanation, Rodolpho disappeared rapidly in the woods leaving the scientists coming back to the base camp with the help of a second guide. This allowed them to rest, eat some food and wait for their impetuous guide. They started to wait a little, a lot… quite a long time… and, then, too long in their opinion. Finally, they got no choice but to spend the night in the base camp, where they received no further news from Rodolpho.

The next day, scientists gradually became more and more impatient, worried, anxious and finally somewhat frightened. They decided to take their way back, convinced that a somewhat “unscrupulous” guide had abandoned them. They convinced the assistant guide to bring them back to town before the end of the day and, late at night, they informed the police of the unexpected and disturbing behavior of the “so experienced” guide they had hired, but who had disappeared the day before. As the adventurous side of Rodolpho was known in town, the searches begun only a few days later when it became obvious that something wrong had happened in the forest. The assistant guide led the police to the base camp where they found no sign of life, and agreed that Rodolpho had not been back to the last place where he was seen. Five days later, several military people were sent into the forest, but they were unfortunately unable to track Rodolpho. Ten to 12 days later a helicopter was sent hovering over the forest, but after several hours of active search, the pilots returned without further news. Therefore, 12 days after his disappearance, Rodolpho was considered as definitively lost and probably dead somewhere in the vastness of the forest.

The news of Rodolpho’s disappearance took some time to reach Grenoble, in the south of France, where it arrived along with the announcement of the cancellation of searches. Andrew, who did consider Rodolpho an experienced forest guide and not likely to be a victim of the jungle tricks, was first devastated to learn that his longtime friend could have died in the forest. At the same time, he could not believe and get used to the idea that Rodolpho had mysteriously disappeared and was lost in the forest when he knew so well the dangers. He quickly decided to check the situation by himself, to leave his company in France, to cross the Atlantic by air and to reach the place where Rodolpho was seen for the last time.

Andrew managed to phone French Guiana and, within hours, a message was sent to a common friend in charge of calling for the invaluable contribution of the three best native indigenous trackers of the village that Rodolpho and him knew so well. Then, Andrew jumped on a plane and, less than 48 h after receiving the news in France, he was heading to the forest with three men he knew for their exceptional skills of trackers and was starting to seek Rodolpho.

Indigenous trackers are exceptionally gifted to find the smallest traces in the forest and these ones were perfectly trained for this search. Quite unfortunately, the trackers had to unravel the countless footsteps of different people who trampled the site in search of Rodolpho. However, once more to Andrew’s great wonder, they managed to find a tiny indication of the likely passage of Rodolpho who, two weeks earlier, had the good sense to leave small traces of cuts on a few lower branches. Having found this fragile thread that had escaped the others, they walked cautiously and slowly to find more clues. After a few hundred meters, they found a few other imperceptible signs that led them to a natural steep ditch in which they went down carefully.

In this specific location, the trackers were adamant that there were almost erased traces still visible to them (but not to Andrew) and they suggested that the person who had fallen in this sort of natural pit had been fighting against the brambles and cutting vegetation in a disorganized way. Tracks then led them throughout the natural slope of the ditch that was moving inexorably away from the base camp. Further, the first traces of survival were evident to Andrew himself as a cluster of tree leaves carefully stacked in a depression where Rodolpho had probably spent a night. From a litter of leaves to another, the trackers were trying to move in the footsteps of Rodolpho, aware of his progressive physical degradation: the space between the litters gradually decreased and no trace of branch cut was anymore detectable. Nevertheless, they found the remnant of a butchered ground turtle and agreed that this animal had been eaten by a human being.

Further, they found traces of a person who crawled on the forest ground, likely exhausted and unable to walk and the trackers suddenly had a lively discussion because they could also identify the pugmarks of a big cat, a jaguar, above these human traces. Finally, some 60 m from the last litter, they saw a body lying on the ground and discovered Rodolpho immobile, severely emaciated, thin, in pain and shivering but still alive.

There was an urgent need to act quickly and carefully and Rodolpho was immediately brought back to the hospital in French Guyana where he was appropriately treated with antibiotics and intravenously rehydrated and fed to recover from his loss of weight. All local French journals announced how Andrew and his team of indigenous trackers successfully located their old friend, almost moribund, dehydrated, feverish, covered with bug bites all over his body, with a deeply infected wound in his left hand and many pounds slimmer than 16 days before.

When appropriately rehydrated, fed and psychologically assisted, Rodolpho managed to explain his astonishing story. Once alone in search of the poisonous frogs, Rodolpho went into the forest with agility and speed. He was deftly wielding his cutlass and went cutting what should be a temporary trail. Unfortunately, for a small but dramatic second, his left hand suddenly slipped off the thorns (made slippery by the recent rains) and at the same time, his cutlass ricocheted off a vine cutting his thumb and part of his left hand with the sharp tool. The cut was deep and bleeding abundantly. He immediately harvested a few leaves of a plant with “antiseptic properties” and put them on the wound covering it in an improvised tight bandage with a strip of cloth^3^. Annoyed, he then continued to walk rapidly and after some distance, he spotted the first two specimens of frogs he was looking for. He managed to pick one of them with his left hand that he considered protected by the layers of plants and cloth^4^. After this successful capture, he did not walk much, before being surprised by a fine rain that quickly turned into a torrential one that soaked Rodolpho, dripping on the frog and then on the bandage. He should have been more concerned by his injury.

Unfortunately, he did not realize that he was walking with the poisonous animal in his injured and insufficiently protected hand. The waterlogged bandage no longer represented an efficient barrier to the poison that, instead, diffused into the cloth penetrating through the wound and spreading in his body.

With the circulating poison, Rodolpho rapidly went into a sort of semi-conscious trance during which he continued walking without noticing that he was losing his usual sense of direction. He was unable to determine for how long he walked with a sort of altered consciousness, then he became almost unconscious until he felt comatose and ended up falling into some kind of ditch he had not seen and in which he struggled before collapsing. When he awoke, probably hours later, he remained unable to locate himself and return to the camp. He decided to follow the slope of the pit when he realized that his appeals would remain with no effect. Still semi-conscious, unable to appropriately organize his thoughts, he walked unsteadily into the forest towards what he hoped to be the river, but he soon discovered that he was facing a kind of poorly defined flooded area and decided to go in one single direction. Unfortunately, he chose the one that led him away from the river and the base camp.

Despite his lack of lucidity, he was decided not to die on the spot and forced himself to walk, thinking that someone would come looking for him. But hours and days passed without seeing anyone, and when a helicopter flew away from him, he realized that he had only little chance of surviving out of this adventure. Even so, he managed to find a turtle that he struggled to kill, and ate raw despite its horrible taste, and this meal – with some rotten fruits found on the ground and a few roots – probably saved his life. Over the last two days of his trial, whereas he was crawling painfully on the floor, unable to stand up and walk, he noticed, at a distance, the presence of a jaguar apparently intrigued by the unusual show he offered unintentionally. The most terrible had been the rains and cold at night when he tried to sleep, always badly, covered with wet leaves, which invariably attracted stinging insects that woke him up every minute. But even so he was determined to survive.

Once saved, Rodolpho was aware of the extraordinary opportunity to have developed with Andrew a unique friendship with almost no limits. He was fortunate to be the friend of a man capable of instantly abandoning the comfort of his home and his business to go in the desperate search of an old friend considered officially dead on the other side of the Atlantic. Rodolpho was also totally indebted to his other friends, the local trackers he knew for having traveled and hunted with them. They dropped everything to come looking for him and managed so efficiently to track and find him so far from their village and so deep into the rain forest, in a place from where he would have never been able to come back on his own.

Andrew was asked to come for an exclusive interview with Rodolpho but kindly refused, saying “There are things that only the sky must watch… ”. Rodolpho passed away several years later and since the “frog episode”, Andrew continued to visit the village of his indigenous friends every year.

## Conclusions

Driven by their enthusiasm and thirst for discovery, scientists are capable to undertake long journeys in remote forested areas of foreign countries and by doing so they tend to take obvious risks for their own safety when they venture into unknown and potentially hostile environments. Indeed, if they are careful enough, they call on the help of specialists who will safely guide them, but they can also involuntarily lead their guide in dire situations. This is precisely what almost cost the life of an experienced and serious naturalist guide.

The friendships that men sometimes forge over time can come to light with dramatic intensity in amazing circumstances. This is what impressed us the most in the true facts that we describe herein to the best according to our now distant but still well alive memories of those unusual events. Even if this is not strictly speaking a scientific report, we consider that it is a unique and heartwarming story in which are entangled frog toxins, pitfalls of the great rainforest and a form of rare brotherhood expressed at the highest degree, that deserves, in our opinion, to be reported to readers.

## Footnotes

1 One of the authors (CR) knew personally the characters, was with them in the hospital just after Rodolpho was rescued from the forest, and could verify the coincidence of their storylines. Although we do not always have explanations for all possible details that may rise from this tale, we believe that it is a factual narrative.

2 Fictitious names of the characters.

3 One of the authors (CR) does remember the longitudinal cut on Rodolpho’s thumb when he visited him at the hospital.

4 Rodolpho was used to manipulate most wild animals, even venomous ones, bare hands and having left the scientists quickly he had not asked for a suitable bag to enclose his catch.
